# Psychostimulant effects on motor and cognitive function in adults attention deficit hyperactivity disorder

**DOI:** 10.1093/ijnp/pyag013

**Published:** 2026-03-26

**Authors:** Maurizio Cundari, Lukas Kanold, David Bergwall, Susanna Vestberg, Amelia Hansson, Lena Kirchhoff, Peik Gustafsson, Anders Rasmussen

**Affiliations:** Department of Experimental Medical Science, Faculty of Medicine, Lund University, Lund, Sweden; Unit of Neurology, Hospital of Helsingborg, Helsingborg, Sweden; Unit of Neuropsychiatry, Hospital of Helsingborg, Helsingborg, Sweden; Department of Experimental Medical Science, Faculty of Medicine, Lund University, Lund, Sweden; Department of Experimental Medical Science, Faculty of Medicine, Lund University, Lund, Sweden; Department of Psychology, Faculty of Social Science, Lund University, Lund, Sweden; Department of Psychology, Faculty of Social Science, Lund University, Lund, Sweden; Department of Psychology, Faculty of Social Science, Lund University, Lund, Sweden; Child and Adolescent Psychiatry, Department of Clinical Sciences Lund, Medical Faculty, Lund University, Lund, Sweden; Department of Experimental Medical Science, Faculty of Medicine, Lund University, Lund, Sweden

**Keywords:** ADHD, neuropsychiatry, neurocognition, neuropsychopharmacology, sensorimotor integration, visuospatial perception, visuospatial abilities, cerebellum

## Abstract

**Objectives:**

This study aims to compare the performance on motor and cognitive functions of adult patients with attention deficit hyperactivity disorder (ADHD) with or without psychostimulant medication and controls. A secondary objective is to investigate differences on test performance across varying levels of disorder severity.

**Methods:**

We included patients with unmedicated ADHD (U-ADHD) (*n* = 52), patients with ADHD treated with psychostimulants (*n* = 40), and controls (*n* = 80). A multimodal set of motor and cognitive tests was administered to evaluate cerebellar and motor functions, attention and processing speed, executive functions, visuospatial perception, and visuospatial abilities.

**Results:**

Both patient groups performed significantly worse than controls across all functions. No significant performance differences were observed between the medicated and U-ADHD groups when disorder severity was not considered. However, some differences emerged when stratified by disorder severity. Patients with moderate to severe U-ADHD showed more impairments in sensorimotor functions compared to the medicated ADHD (M-ADHD), while those with mild U-ADHD displayed lower scores on visuospatial perception compared to M-ADHD. Regression analysis indicated that education, anxiety, and sleep disturbances minimally affect test performance.

**Conclusions:**

In summary, psychostimulant medication did not show consistent overall differences in motor and cognitive performance among adults with ADHD. Importantly, when stratifying by disorder severity, some group-level differences emerged, underscoring the need to account for disorder severity in future research and clinical assessment.

## Introduction

Attention deficit hyperactivity disorder (ADHD) is one of the most common neurodevelopmental disorders^1–3^ with a prevalence of 5.3% among children and adolescents,^4^ and 2.5% among adults.^5^ ADHD is characterized by impulsivity, hyperactivity, and inattentiveness and is often associated with impairments in social, academic, and occupational functioning. ADHD can be categorized by severity into mild, moderate, and severe based on the number of excess criteria fulfilled and to what extent life aspects are impaired.^6^ ADHD is typically diagnosed via assessment by clinicians and parent-teacher questionnaires.^7^ Previous studies suggest that there may be a risk of subjectivity to these methods,^8^ but no biomarkers or behavioral markers have proven sufficient to replace them.^7^ ADHD affects patients’ lives in a multitude of ways, including poor academic performance,^9^ lower socioeconomic status,^10^ and a shortened life expectancy.^11^ Individuals with ADHD often have additional comorbidities, like anxiety, depression and sleep disorders, further exacerbating the difficulty of ADHD patients’ lives.^12^

Pharmacological treatment has proven effective at reducing ADHD symptoms,^13^ and has displayed great potential in improving patients’ life outcomes.^14^ Medicine options for ADHD include psychostimulants such as amphetamine and methylphenidate as well as nonstimulants such as guanfacine and atomoxetine.^15^ Psychostimulants are an effective treatment for ADHD,^13^ and methylphenidate is currently considered the first-line treatment in patients aged 5 years and older.^15^

Psychostimulants enhance activity in the prefrontal cortex, thereby improving attention and executive functions, by increasing extracellular dopamine and norepinephrine, primarily through inhibition of their transporters, which prolongs the presence of these neurotransmitters in the synaptic cleft.^16^^,^^17^ When first-line treatment is unsuitable due to severe side effects^18^ or lack of response,^19^ nonstimulant agents may be used as alternatives. Guanfacine is a norepinephrine receptor agonist^20^ and atomoxetine is a norepinephrine reuptake inhibitor,^21^ both of which significantly reduce ADHD symptoms.^22^

Several cognitive functions, such as executive functions, attention and processing speed, visuospatial perception and abilities, are adversely affected in individuals with ADHD.^23–26^ The cognitive profile of ADHD patients depicted in the Wechsler Adult Intelligence Scale IV (WAIS-IV) describes a substantial deficit in processing speed and working memory.^27^ Previous research suggests that processing speed affects general intelligence and higher cognitive functions.^28^ Visuospatial abilities play an integral role in processing and mentally manipulating visual and spatial information from our environment,^29^ which is important for performing a wide range of tasks, and is required for high-level performance in many fields, such as the musical, athletic, and artistic disciplines.^30^ It has been suggested that motor skill deficiencies are present in most children with ADHD.^24^ Furthermore, neuroimaging studies propose that there is diminished activation in brain regions important for attention, emotion regulation, and sensorimotor functions in ADHD patients.^31^

The cerebellum plays an integral role in several cognitive domains including sensorimotor function, executive functions, emotional processing, and visuospatial abilities.^32^ Previous research suggests that ADHD patients exhibit difficulties in tasks dependent on cerebellar functions.^26^^,^^33^ Executive functions, attention and processing speed, visuospatial abilities, and sensorimotor function contribute to a wide range of cognitive processes that could be important for academic and occupational performance as well as everyday life.^28^^,^^34–36^ Exploring how commonly prescribed ADHD medication, such as psychostimulants, affects their test results can provide further insight into how patients cognitive functions are affected by medication. Furthermore, investigating how ADHD patients perform on cognitive tests may provide valuable information that could potentially be used for diagnostic purposes and improving clinical assessments.

This study aims to compare the performance on motor and cognitive functions of adult patients with Attention Deficit (ADHD) with or without psychostimulant medication, with controls and with each other (1). In addition, we assess whether varying degrees of disorder severity impact test performance and explore potential interaction effects between severity and medication status (2). Specifically, we assess cerebellar and motor functions, visuospatial perception and visuospatial abilities, attention and processing speed, and executive functions. We explore if patients medicated with psychostimulant have reduced symptoms for anxiety, depression, and sleep (3). We hypothesize that unmedicated ADHD (U-ADHD) patients will perform significantly worse than medicated ADHD (M-ADHD) patients, and that test performances will be significantly lower in individuals with ADHD compared to controls.

## Methods

### Study design

This was a cross-sectional observational study. The primary variables of interest included outcome measures from tests of cerebellar and motor functions, visuospatial perception and abilities, attention and processing speed, and executive functions, as well as grouping factors such as diagnosis, medication status, and disorder severity. We also examined potential moderating variables including age, education, anxiety, depression, and insomnia. The participants went through a clinical interview to assess their mental and physical health, past health issues, sleep difficulties, language disorders, hereditary conditions, alcohol and drug use, and current medications.

### Standard protocol approvals, registrations, and patient consents

#### Ethical approval and consent to participate

This study was approved by the Swedish Ethical Review Authority (Dnr 2022-01799-01). Participants showing signs of mental disorder, suicidal thoughts, or concerning test results were referred to appropriate care. All the human data in the study were collected in line with the 1964 Helsinki Declaration. Furthermore, the data collected were handled in accordance with the Swedish Patient Data Act (2008:355) as well as the General Data Protection Regulation.

#### Consent to publication

All participants were informed that the results would be published but that they would remain anonymous. The manuscript contains no information that can lead to the identification of the participants.

### Selection and description of the participants

A total of 257 adults, 156 patients and 101 controls, were tested between 2023 and 2025. The patients were recruited from the Unit of Adult Neuropsychiatry from Helsingborg’s hospital, Sweden. We included patients with U-ADHD and M-ADHD. The ADHD severity index was assessed based on Diagnostic and Statistical Manual of Mental Disorders, Fifth Edition criteria (1–Mild, 2–Moderate, or 3–Severe) and was determined before the initiation of medication.^6^ The severity was based before the initiation of medication. The inclusion criteria were: (1) age between 18 and 75, (2) ability to speak Swedish, and (3) a diagnosis of ADHD documented in their medical journal. Exclusion criteria were: previous brain injury, intellectual disability, and continuous substance abuse. We excluded 2 patients with undiagnosed intellectual disability, detected during neurocognitive assessment; 6 patients who showed neuropsychiatric symptomatology but did not fulfill diagnostic criteria, 1 patient with ADHD and cardiovascular-related brain injury, 1 patient with autism spectrum disorder (ASD) medicated with methylphenidate, 23 patients medicated for ADHD and who also had an ASD diagnosis and 31 patients unmedicated for ADHD and who also had an ASD diagnosis. The final sample consisted of 92 patients. Comorbidity and medication details are in Supplementary Materials S1 and S2. For the controls, the inclusion criteria were: (1) age between 18 and 75, and (2) ability to speak Swedish. Controls were excluded if they had a psychiatric diagnosis, neurological disorder, intellectual disability, previous brain injury, sleep disorder, language impairment, or continuous substance abuse. Of the 101 controls, 95 fulfilled the inclusion criteria. We also excluded all control participants older than 58 to match the age range of the patient groups. Table 1 shows the distribution of participants included in the final analysis.

**Table 1:** Demographic and clinical characteristics.

**Characteristics**	**Controls** **(*n* = 80)**	**U-ADHD** **(*n* = 52)**	**M-ADHD** **(*n* = 40)**	***P***
Sex Male, female	43; 37	21; 31	13; 27	.351
Age Years	31.5 (25-43.3) [20-58]	29 (21.3-35.8) [18-58]	26.5 (21-38.5) [18-55]	.060
Education Years	16 (14-16) [10-23.5]	14 (13-15) [10-18]	13 (13-16) [10-26]	**<.001**
Anxiety Scores	5 (3-7) [0-11]	13 (10-15) [3-18]	9 (6-13) [2-18]	**<.001**
Depression Scores	2 (1-3.8) [0-9]	7 (4-10) [1-15]	5 (3-6) [0-20]	**<.001**
Sleep Scores	5.5 (2-8) [0-17]	13 (9-17) [2-23]	12 (7-16) [0-25]	**<.001**
Severity index 1 Frequency, %	n.a.	32; 61.5%	25; 62.5%	n.a.
Severity index 2	n.a.	17; 32.7%	9; 22.5%	n.a.
Severity index 3	n.a.	3; 5.8%	6; 15%	n.a.
Stratification				
Group 1				
Severity index 1	n.a.	32; 61.5%	25; 62.5%	1.00†
Group 2				
Severity index 2 + 3	n.a.	20; 38.5%	15; 37.5%	.214†

Medians, interquartile ranges, minimum, and maximum values are reported. Kruskal-Wallis was applied for all test variables. Daggers (†) indicate *P*-values calculated using the Mann-Whitney test.

Bold values indicate statistically significant differences.

Abbreviations: Group 1, consists of ADHD patients with mild disorder severity; Group 2, includes those with moderate to severe disorder severity; M-ADHD, medicated ADHD patients; n.a., not applicable; *n*, the sample size for each group; U-ADHD, unmedicated ADHD patients.

### Cerebellar functions

#### Finger tapping

This test measures sensorimotor synchronization and the ability to maintain rhythm and can be used to assess motor control and the integrity of the neuromuscular system.^37^ We evaluated isochronous serial interval production^38^^,^^39^ that has been linked to cerebellar function.^40^^,^^41^ The variables selected for the analysis were production mean (ms), local (ms), and drift (ms). For details see Supplementary Material S3.

#### Prism adaptation

This test measures sensorimotor coordination following changes in visual input. Previous studies demonstrate a relationship between performance in prism adaptation and cerebellar function.^42^^,^^43^ For the analysis, we examined the error in centimeters on the first trial after putting the glasses on (trial 11), the error on the first trial after taking the glasses off (trial 21), and the average absolute error across all 30 trials. For details see Supplementary Material S3.

### Motor functions

#### Motor speed, trail making test of Delis-Kaplan Executive Function System

This subtest measures motor speed without cognitive load. It provides a direct, isolated measure of motor demands.^33^^,^^44^ The task involves precise line-tracing, which taps into fine motor coordination and manual dexterity.

#### Motor test, Bender-Gestalt II

This supplemental test measures fine motor abilities.^45^ As an explorative measure, we also recorded the time it took to complete the task in seconds, we created a variable Motor Time.^33^ For details of psychometric properties see Supplementary Material S4.

#### Motor screening task, Cambridge Neuropsychological Test Automated Battery

All Cambridge Neuropsychological Test Automated Battery (CANTAB) tasks were administered on an iPad (9^th^ generation; 10.5-inch screen).^46^  *Motor Screening Task* measures sensorimotor functions and processing speed. Outcome measures were speed of response and accuracy of pointing. It is used as a basic measure for motor abilities.^47^ The variable selected for the analysis was the mean latency from the display of a stimulus to a correct response to that stimulus (MOTML).

### Cognitive assessments for visuospatial perception and visuospatial abilities

#### Cube analysis and silhouettes, subtests of visual object and space perception battery

Cube analysis measures visuospatial perception and visual discrimination. Silhouettes measure visuospatial perception and the ability to recognize common objects and animals depicted from unconventional perspectives.^48^

#### Copy task, visual reproduction II of Wechsler Memory Scale III

The participants were instructed to copy 5 figures, presented one at a time. Each stimulus was available to the participant continuously, thus, removing the memory aspect and focusing more on visuospatial abilities and visuomotor integration.^49^ Participants were instructed to quickly and accurately reproduce the figure and inform the test administrator when finished. We recorded the time it took to copy each figure. Scoring of the 5 figures was done in accordance with the Wechsler Memory Scale III manual.^50^ The criteria measure accuracy and spatial positioning.

### Cognitive assessments for attention and processing speed

#### Coding and symbol search, subtests of WAIS-IV

Both subtests measure sensorimotor integration, visual scanning, and processing speed. The *Coding* test primarily measures incidental learning and visual-motor coordination, whereas the *Symbol Search* test primarily measures visual discrimination and visual scanning.^27^^,^^51^

#### Perception test, Bender-Gestalt II

This supplemental test measures visual scanning and perceptual abilities.^45^ As an explorative measure, we also recorded the time it took to complete the task in seconds, we created a variable Perception Time.^33^ For details of psychometric properties see Supplementary Material S4.

#### Visual scanning, trail making test of Delis-Kaplan Executive Function System

This subtest measures visual scanning ability, visual, and selective attention. This specific condition isolates visual scanning ability by removing the need for motor sequencing or number-letter alternation.^44^

#### Reaction time, CANTAB

*Reaction time* (RTI) measures processing, psychomotor speed, and movement.^46^ Measures include RTI and movement time for both the simple and 5-choice modes. The variables selected for analysis were RTIFMRT (the mean time taken for a subject to release the response button after the presentation of a target stimulus), and RTIFMMT (the mean time taken for a subject to select the target stimulus after releasing the response button). These variables are measured in milliseconds.

### Cognitive assessments for executive functions

#### Number sequencing and letter sequencing, trail making test of Delis-Kaplan Executive Function System

These subtests assess number and letter sequencing ability, which requires working memory and visuomotor tempo, and is a component skill necessary to complete the Trail Making Test Condition 4—Number-Letter Switching.^44^

#### Number-letter switching, trail making test of Delis-Kaplan Executive Function System

This subtest primarily measures cognitive flexibility, while also measuring inhibition ability and visuomotor tempo.^44^ Beyond primarily assessing cognitive flexibility, it also evaluates inhibitory control, visuomotor speed, working memory, and task-switching efficiency.

#### Inhibition, color-word interference test of Delis-Kaplan Executive Function System

The test has 4 conditions, of which the third was of interest in this study. This condition^44^ requires the inhibition of the learned behavior of reading words aloud as they are written. The variable selected for the analysis was total time in seconds.

### Clinical Scales

#### Hospital Anxiety and Depression Scale

The Hospital Anxiety and Depression Scale (HADS) is a self-report questionnaire that contains 14 items, 7 items measuring cognitive and emotional aspects of depression, specifically anhedonia, and the remaining 7 items focusing on cognitive and emotional aspects of anxiety.^52^^,^^53^ High scores indicate greater severity. Both HADS-A (α = .87) and HADS-D (α = .81) have high reliability.

#### Insomnia Severity Index

The Insomnia Severity Index (ISI) is a 7-item self-report questionnaire assessing insomnia severity and impact in the last 2 weeks.^54^ High scores indicate greater severity. ISI has high reliability (α = .79).

### Statistics

All analyses in this study were performed using IBM SPSS Statistics version 28.0. The results are reported as median and interquartile range (Q1-Q3) for non-normally distributed variables, and as mean ± SD for normally distributed variables. For parametric analyses, normality was evaluated using the Shapiro-Wilk test, and homogeneity of variances was assessed with Levene’s test. When assumptions for parametric testing were met, analyses of variance (ANOVA) were applied. Outlier exclusion was performed only for variables using ANOVA, based on predefined distributional criteria. When normality assumptions were not satisfied, non-parametric tests were used. Comparisons within disorder severity groups were conducted using the Mann-Whitney U test. To reduce the risk of type I error due to multiple testing, Bonferroni correction was applied to all the test variables. Patients were divided into 2 groups based on disorder severity, mild ADHD (Group 1), and moderate to severe ADHD (Group 2). Linear and multiple regression were used to control if years of education, anxiety, depression, and sleep disturbances affected test performance. Education was consistently included as a predictor for all outcomes, whereas anxiety, depression, and sleep disturbances were examined for all the significant outcomes.

## Results

### Participants

Table 1 shows the distribution of demographics and clinical variables. A Kruskal-Wallis analysis showed that there were small but significant differences in years of education but not in sex or age. Post hoc analysis revealed significant differences in years of education between controls and U-ADHD (*P* = .003) and between controls and M-ADHD (*P* = .001).

### Cerebellar and motor functions

Table 2 provides an overview of performances. After Bonferroni correction, we found significant differences between controls and the 2 patient groups for 6 variables*.* The results showed differences in performance with large effect size on *Prism Error Mean* and *Motor Speed* and differences with medium effect size on *Finger Tapping Production Mean, Motor Time, Prism First With* and *Prism First Without.* No significant differences were found for *Finger Tapping Local Variation* (*P* = .149), *Finger Tapping Drift* (*P* = .326) or MOTML of *Motor Screening Task* (*P* = .559). Post hoc analysis revealed no significant differences between the 2 patient groups.

**Table 2:** Descriptive statistics for the 3 groups on test performances of cerebellar and motor functions.

**Measure**	***n***	**Controls**	***n***	**U-ADHD**	***n***	**M-ADHD**	***H***	***P***	***η*** ^**2**^
Production mean ms	73	517 (503-524) [467-556]	51	501 (478-510) [395-559]	40	498 (488-511) [459-566]	21.23	**<.001**	0.119
Local ms	75	24 (20-28) [16-50]	48	26 (22-31) [17-47]	39	28 (21-34) [14-67]	3.81	.149	0.011
Drift ms	74	7 (4-10) [0-25]	51	7 (4-16) [0-40]	39	8 (4-14) [0-28]	2.24	.326	0.002
Prism first with cm	80	9 (4.6-13) [−5 to 28]	52	12 (9-15) [3-22]	40	12 (11-15.8) [5-22]	16.02	**<.001**	0.083
Prism first without cm	80	−5 (−6.4 to (−3)) [−13 to (−3)]	52	−6 (−8 to (−5)) [−15 to (−7)]	40	−7 (−8 to (−5)) [−14 to (−3)]	14.54	**<.001**	0.074
Prism error mean cm	80	2.1 (1.5-2.6) [0.5-4.8]	52	3 (2.5-3.7) [1.4-7.1]	40	3.3 (2.6-4.1) [2.1-6]	59.32	**<.001**	0.339
Motor speed sec	80	27 (21-40) [9-70]	52	44.5 (34-79) [17-150]	40	48.0 (36-62) [19-106]	43.43	**<.001**	0.245
Motor time sec	80	44 (36-54) [24-87]	52	53 (45-70) [28-87]	40	52 (44-64) [26-83]	20.89	**<.001**	0.113
MOTML ms	80	705 (611-861) [490-1384]	52	748 (627-897) [542-1459]	40	735 (653-848) [491-1189]	1.56	.457	0.005

Medians, interquartile ranges, minimum, and maximum values, *H*-statistic values, and effect sizes for the Kruskal-Wallis test are reported. Sample sizes differ across groups due to the exclusion of outliers. Data for the *Finger Tapping* test *(production mean, local and drift)* are missing due to problems with the device.

Bold values indicate statistically significant differences.

Abbreviations: cm, centimeters; M-ADHD, medicated ADHD patients; MOTML, the mean latency from the display of a stimulus to a correct response to that stimulus; ms, milliseconds; *n*, the sample size for each group; U-ADHD, unmedicated ADHD patients.

### Cognitive functions

#### Visuospatial perception and visuospatial abilities

Table 3 shows all the cognitive test performance. None of the participants scored below 15 on *Shape Detection Screening Test* of *Visual Object and Space Perception Battery.*^34^ The results showed significant differences with large effect size on *Cube Analysis* and *Copy Task* and differences with medium effect size on *Silhouettes.* No significant differences were found for total time of *Copy Task* (*P* = .072). After Bonferroni correction, we found significant differences between controls and the 2 patient groups for *Cube Analysis*, *Copy Task*, and *Silhouettes.* No significant differences emerged between the 2 patient groups.

**Table 3:** Descriptive statistics for the 3 groups on test performances of cognitive functions.

**Measure**	**Cognitive function**	**Neurocognitive domain**	***n***	**Controls**	***n***	**U-ADHD**	***n***	**M-ADHD**	***H***	***P***	***η*** ^**2**^
**Cube analysis** rs	Visuospatial perception	**Visual perception and visuospatial abilities**	78	10 (10-10) [8-10]	52	9 (8-10) [3-10]	40	9 (9-10) [7-10]	44.97	**<.001**	0.259
**Silhouettes** rs	Visual perception	NA	80	23 (21-26) [12-29]	52	20 (17-23) [11-30]	40	21 (18-23) [15-28]	22.02	**<.001**	0.118
**Copy task** rs	Visuospatial abilities	NA	80	93 (90-98) [80-104]	52	88 (84-92) [68-100]	40	89 (86-92) [75-102]	35.71	**<.001**	0.199
**Copy task** sec	Visuospatial abilities	NA	80	130 (104-167) [52-311]	52	146 (117-227) [74-382]	40	133 (101-178) [73-327]	3.27	.019	0.020
											
**Coding** rs	Visual discrimination	**Attention and** **processing speed**	80	75 ± 12.2 [39-100]	52	57.4 ± 13.8 [25-92]	40	58.3 ± 15.4 [25-94]	34.99†	**<.001** ^**a**^	0.293
**Symbol search** rs	Visual scanning	NA	80	38 ± 8.9 [16-68]	52	29.9 ± 8.4 [11-51]	40	30.8 ± 8.5 [13-49]	19.59†	**<.001** ^**a**^	0.188
**Perception time** sec	Visual scanning	NA	80	19 (17-23) [11-36]	52	30 (25-37) [16-68]	40	27 (24-34) [17-50]	65.27	**<.001**	0.374
**Visual scanning** sec	Selective attention	NA	80	18 (15-21) [12-30]	52	23 (19-26) [15-47]	40	20 (18-23) [14-26]	35.35	**<.001**	0.198
**RTIFMRT** ms	Psychomotor speed	NA	79	382 ± 42 [304-528]	52	403 ± 35 [337-512]	39	385 ± 40 [311-462]	4.56†	**.012** ^**a**^	0.052
**RTIFMMT** ms	Psychomotor speed	NA	80	233 (201-279) [107-475]	52	277 (237-323) [144-500]	40	252 (225-299) [157-456]	12.73	**.002**	0.064
**Number** **sequencing** sec	Sequencing ability	**Executive** **functions**	80	30 (24-35) [15-56]	52	34 (28-44) [21-71]	40	33 (27-44) [18-68]	9.53	**.009**	0.042
**Letter** **sequencing** sec	Sequencing ability	NA	80	29 (24-35) [13-57]	52	32 (25-50) [20-93]	40	32.0 (22-39) [17-55]	6.36	**.041**	0.060
**Number-letter switching** sec	Cognitive flexibility	**NA**	80	61 (52-75) [36-124]	52	99 (68-140) [48-240]	40	92 (63-132) [39-240]	38.41	**<.001**	0.215
**Inhibition** sec	Inhibition ability	NA	80	45 (39-51) [31-77]	52	58 (47-72) [35-97]	40	64 (45-72) [30-126]	43.83	**<.001**	0.250

The mean, SD, minimum, and maximum values, effect sizes, *F*-statistic values for ANOVA, and medians, interquartile ranges and *H*-statistic values for the Kruskal-Wallis test are reported. Sample sizes vary across groups due to the exclusion of outliers. “a” indicates *P*-values calculated using the ANOVA, while Kruskal-Wallis test was used for all other variables. Data from 2 control participants were missing for the *Cube Analysis* due to incorrect test administration. In addition, one control participant and one M-ADHD participant were excluded from the *RTIFMRT* analysis due to extreme values. Daggers (†) denote *F*-statistic values for the ANOVA.

Bold values indicate statistically significant differences.

Abbreviations: M-ADHD, medicated ADHD patients; ms, milliseconds; *n*, the sample size for each group; NA, not available; rs, raw scores; RTIFMMT, the mean time taken for a subject to select the target stimulus after releasing the response button; RTIFMRT, the mean time taken for a subject to release the response button after the presentation of a target stimulus; sec, seconds; U-ADHD, unmedicated ADHD patients.

#### Attention and processing speed

The results showed significant differences with a large effect size on *Coding, Symbol Search, Perception Time,* and *Visual Scanning,* differences with medium effect size on *RTIFMMT* and differences with small effect size on *RTIFMRT.* After Bonferroni correction, we found significant differences between controls and the 2 patient groups for all variables. No significant differences emerged between the 2 patient groups.

#### Executive functions

The results showed significant differences with large effect size on *Number-Letter Switching* and *Inhibition*, medium effect size on *Letter Sequencing*, and differences with small effect size on *Number Sequencing.* After Bonferroni correction, significant differences between controls and patient groups remained for all variables. However, when examined separately, the only significant difference between controls and the U-ADHD group was observed for *Letter Sequencing* (*P* = .035).

### Exploratory analyses: motor and cognitive function outcomes stratified by severity index

#### Severity index 1—Group 1

The severity index distribution of patients in the 2 stratified groups is shown in Table 1. Exploratory analyses of motor and cognitive outcomes stratified by severity index are presented in Table 4. After Bonferroni correction, significant differences were observed between the control group and both patient groups. For *Production Mean*, significant differences were observed between controls and M-ADHD (*P* = .008) and between controls and U-ADHD (*P* = .030). For *Prism First With*, significant differences were observed between controls and M-ADHD (*P* = .006) and between controls and U-ADHD (*P* = .047). For *Prism Error Mean*, significant differences were observed between controls and M-ADHD (*P*<.001) and between controls and U-ADHD (*P*<.001). For *Motor Speed*, significant differences were observed between controls and M-ADHD (*P*<.001) and between controls and U-ADHD (*P*<.001). For *Motor Time*, significant differences were observed only between controls and the U-ADHD group (*P* = .006).

**Table 4:** Exploratory analyses of motor and cognitive outcomes stratified by severity index.

**Outcome**	**Group 1 (Severity index 1)**	**Group 2 (Severity index 2-3)**
**Production mean**	C vs M-ADHD (*P* = .008) C vs **U-ADHD** (*P* = .030)	C vs M-ADHD (*P* = .013) C vs **U-ADHD** (*P* = .001)
**Prism first with**	C vs M-ADHD (*P* = .006) C vs **U-ADHD** (*P* = .047)	C vs **U-ADHD** (*P* = .048)
**Prism first without**	–	C vs M-ADHD (*P* = .013)
**Prism error mean**	C vs M-ADHD (*P*<.001) C vs **U-ADHD** (*P*<.001)	C vs M-ADHD (*P*<.001) C vs **U-ADHD** (*P*<.001)
**Motor speed**	C vs M-ADHD (*P*<.001) C vs **U-ADHD** (*P*<.001)	–
**Motor time**	C vs **U-ADHD** (*P* = .006)	–
**Cube analysis**	C vs M-ADHD (*P* = .002) C vs **U-ADHD** (*P*<.001)	C vs M-ADHD (*P*<.001) C vs **U-ADHD** (*P* = .013)
**Silhouettes**	C vs M-ADHD (*P* = .008) C vs **U-ADHD** (*P* = .011)	C vs **U-ADHD** (*P*<.001)
**Copy task**	C vs M-ADHD (*P*<.001) C vs **U-ADHD** (*P* = .003)	C vs **U-ADHD** (*P*<.001) M-ADHD vs **U-ADHD** (*P* = .002)
**Copy task (time)**	–	C vs **U-ADHD** (*P* = .002)
**Coding**	C vs M-ADHD (*P*<.001) C vs **U-ADHD** (*P*<.001)	C vs M-ADHD (*P*<.001) C vs **U-ADHD** (*P*<.001)
**Symbol search**	C vs M-ADHD (*P* = .004) C vs **U-ADHD** (*P*<.001)	C vs M-ADHD (*P*<.001) C vs **U-ADHD** (*P*<.001)
**Visual scanning**	C vs **U-ADHD** (*P*<.001)	C vs M-ADHD (*P* = .006) C vs **U-ADHD** (*P*<.001)
**Perception time**	C vs M-ADHD (*P*<.001) C vs **U-ADHD** (*P*<.001)	C vs M-ADHD (*P*<.001) C vs **U-ADHD** (*P*<.001)
**RTIFMMT**	C vs **U-ADHD** (*P* = .005)	C vs **U-ADHD** (*P* = .058)
**Number sequencing**	–	C vs **U-ADHD** (*P* = .042)
**Letter sequencing**	–	C vs **U-ADHD** (*P* = .002)
**Number-letter switching**	C vs M-ADHD (*P* = .001) C vs **U-ADHD** (*P*<.001)	C vs M-ADHD (*P* = .004) C vs **U-ADHD** (*P*<.001)
**Inhibition**	C vs M-ADHD (*P* = .003) C vs **U-ADHD** (*P*<.001)	C vs M-ADHD (*P*<.001) C vs **U-ADHD** (*P*<.001)

Only statistically significant pairwise comparisons after Bonferroni correction are reported.

Abbreviations: C, controls; M-ADHD, medicated ADHD; RTIFMMT**,** the mean time taken for a subject to select the target stimulus after releasing the response button; U-ADHD, unmedicated ADHD (U-ADHD highlighted in bold); –, indicate no statistically significant differences.

In the *Cube Analysis*, significant differences were found between controls and U-ADHD (*P*<.001) and between controls and M-ADHD (*P* = .002). For *Silhouettes*, significant differences were observed between controls and M-ADHD (*P* = .008) and between controls and U-ADHD (*P* = .011). For the *Copy Task*, significant differences were observed between controls and M-ADHD (*P*<.001) and between controls and U-ADHD (*P* = .003). For *Coding*, significant differences were observed between controls and M-ADHD (*P*<.001) and between controls and U-ADHD (*P*<.001). For *Symbol Search*, significant differences were observed between controls and M-ADHD (*P* = .004) and between controls and U-ADHD (*P*<.001). For *Visual Scanning*, significant differences were observed only between controls and the U-ADHD group (*P*<.001). For *Perception Time*, significant differences were observed between controls and M-ADHD (*P*<.001) and between controls and U-ADHD (*P*<.001). In the *RTIFMMT*, significant differences were observed only between controls and the U-ADHD group (*P* = .005). For *Number–Letter Switching*, significant differences were observed between controls and M-ADHD (*P* = .001) and between controls and U-ADHD (*P*<.001). For *Inhibition*, significant differences were observed between controls and M-ADHD (*P* = .003) and between controls and U-ADHD (*P*<.001).

#### Severity indices 2 and 3—Group 2

The severity index distribution of patients in the 2 stratified groups is shown in Table 1. Exploratory analyses of motor and cognitive outcomes stratified by severity index are presented in Table 4. After applying Bonferroni correction, significant differences were observed between the control group and both patient groups. For *Production Mean*, significant differences were observed between controls and M-ADHD (*P* = .013) and between controls and U-ADHD (*P* = .001). For *Local*, significant differences were observed only between controls and M-ADHD (*P* = .032). For *Prism First With*, significant differences were observed only between controls and U-ADHD (*P* = .048). For *Prism First Without*, significant differences were observed between controls and M-ADHD (*P* = .013). For *Prism Error Mean*, significant differences were observed between controls and M-ADHD (*P*<.001) and between controls and U-ADHD (*P*<.001). For *Silhouettes*, significant differences were observed only between controls and U-ADHD (*P*<.001). For *Cube Analysis*, significant differences were observed between controls and M-ADHD (*P*<.001) and between controls and U-ADHD (*P* = .013). For the *Copy Task*, significant differences were observed between controls and U-ADHD (*P*<.001) and between M-ADHD and U-ADHD (*P* = .002). For *Copy Task (Time)*, significant differences were observed only between controls and U-ADHD (*P* = .002). For *Coding*, significant differences were observed between controls and M-ADHD (*P*<.001) and between controls and U-ADHD (*P*<.001). For *Symbol Search*, significant differences were observed between controls and M-ADHD (*P*<.001) and between controls and U-ADHD (*P*<.001). For *Visual Scanning*, significant differences were observed between controls and M-ADHD (*P* = .006) and between controls and U-ADHD (*P*<.001). For *Perception Time*, significant differences were observed between controls and M-ADHD (*P*<.001) and between controls and U-ADHD (*P*<.001). For *Number Sequencing*, significant differences were observed only between controls and U-ADHD (*P* = .035). For *RTIFMMT*, significant differences were observed only between controls and U-ADHD (*P* = .058). Significant differences were also found between controls and U-ADHD for *Letter Sequencing* (*P* = .002). For *Number–Letter Switching*, significant differences were observed between controls and M-ADHD (*P* = .004) and between controls and U-ADHD (*P*<.001). For *Inhibition*, significant differences were observed between controls and M-ADHD (*P*<.001) and between controls and U-ADHD (*P*<.001).

### Regression analysis

Linear and multiple regression analyses were conducted to control for years of education, anxiety, depression, and sleep disturbances when examining motor and neurocognitive outcomes (Table 5). Years of education exhibited minimal significant associations with some measures of attention and processing speed, as well as executive and motor functions (Table 5). Years of education were not associated with visual perception and visuospatial outcomes. Depression had no effect on performance, whereas anxiety and sleep had a minimal significant impact on few cognitive and motor outcomes (Table 5).

**Table 5:** Results of the linear and multiple regression analysis for the impact of education, anxiety, depression, and sleep on motor and neurocognitive outcomes.

**Variable**	***R*** ^**2**^	***F* value (1, *df*)**	**Predictor**	***β***	***SE***	**95% *CI*** **Lower bound**	**95% *CI*** **Upper bound**	***P***
*Cerebellar and motor functions*								
Production mean	0.010	1.61 (1, 167)	**Education**	.100	1.00	−0.688	3.29	.198
Local	0.058	10.13 (1, 165)	**Education**	−.241	0.517	−2.66	−0.626	**.002**
Drift	0.016	2.66 (1, 165)	**Education**	−.126	0.305	−1.09	0.104	.105
Prism first with	0.004	0.697 (1, 170)	**Education**	−.064	0.187	−0.524	0.213	.405
Prism first without	0.010	1.63 (1, 170)	**Education**	.098	0.112	−0.078	0.364	.203
Prism error mean	0.036	6.27 (1, 170)	**Education**	−.189	0.038	−0.171	−0.020	**.013**
Motor speed	0.023	4.08 (1, 170)	**Education**	−.153	0.881	−3.52	−0.041	**.045**
Motor time	0.030	5.22 (1, 170)	**Education**	−.173	0.601	−2.55	−0.187	**.024**
MOTML	0.004	0.661(1, 170)	**Education**	−.062	7.32	−20.42	8.50	.417
*Visuospatial perception and abilities*								
Cube analysis	0.017	2.82 (1, 168)	**Education**	.129	1.42	−0.014	0.175	.095
Silhouettes	0.004	0.749 (1, 170)	**Education**	.066	0.136	−0.150	0.385	.388
Copy task	0.079	14.60 (1, 170)	**Education**	.281	0.218	0.403	1.26	**<.001**
Copy task, time	0.003	0.505 (1, 170)	**Education**	−.054	2.76	−7.42	3.49	.478
*Attention and processing speed*								
Coding	0.104	19.76 (1, 170)	**Education**	.323	0.508	1.25	3.26	**<.001**
Symbol search	0.126	24.42 (1, 170)	**Education**	.354	0.301	0.892	2.07	**<.001**
Perception time	0.079	14.57 (1, 170)	**Education**	−.281	0.321	−0.185	−0.591	**<.001**
Visual scanning	0.037	6.46 (1, 170)	**Education**	−.191	0.230	−1.03	−0.131	**.012**
RTIFMRT	0.015	2.56 (1, 170)	**Education**	−.122	1.57	−5.61	0.584	.111
RTIFMMT	0.020	3.46 (1, 170)	**Education**	−.141	2.29	−8.79	0.257	.064
*Executive functions*								
Number sequencing	0.065	11.77 (1, 170)	**Education**	−.254	0.394	−2.12	−0.574	**<.001**
Letter sequencing	0.075	13.82 (1, 170)	**Education**	−.274	0.761	−4.33	−1.32	**<.001**
Number-letter switching	0.076	13.91 (1, 170)	**Education**	−.275	1.62	−9.25	−2.84	**<.001**
Inhibition	0.121	23.20 (1, 170)	**Education**	−.347	0.831	−5.64	−2.36	**<.001**
Production mean	0.042	2.40 (3, 163)	**Anxiety**	−.143	0.756	−2.42	0.564	.221
NA	NA	NA	**Depression**	−.047	0.838	−2.03	1.28	.655
NA	NA	NA	**Sleep**	−.038	0.506	−1.18	0.811	.710
Prism first with	0.106	6.64 (3, 168)	**Anxiety**	.298	0.133	0.094	0.620	**.008**
NA	NA	NA	**Depression**	−.170	0.150	−0.546	0.045	.096
NA	NA	NA	**Sleep**	.159	0.090	−0.032	0.322	.108
Prism first without	0.009	0.51 (3, 168)	**Anxiety**	.058	2.08	−3.07	5.13	.621
NA	NA	NA	**Depression**	.042	2.33	−3.70	5.50	.699
NA	NA	NA	**Sleep**	.005	1.40	−2.69	2.84	.959
Prism error mean	0.230	16.75 (3, 168)	**Anxiety**	.202	0.026	0.000	0.101	.052
NA	NA	NA	**Depression**	−.025	0.029	−0.065	0.049	.793
NA	NA	NA	**Sleep**	.340	0.017	0.030	0.099	**<.001**
Motor speed	0.159	10.59 (3, 168)	**Anxiety**	.200	0.62	−0.08	2.35	.067
NA	NA	NA	**Depression**	−.001	0.69	−1.37	1.36	.991
NA	NA	NA	**Sleep**	.240	0.42	0.22	1.86	**.013**
Motor time	0.100	6.26 (3, 168)	**Anxiety**	.160	0.44	−0.24	1.48	.155
NA	NA	NA	**Depression**	.055	0.49	−0.70	1.23	.589
NA	NA	NA	**Sleep**	.144	0.29	−0.15	1.01	.146
Cube analysis	0.092	5.58 (3, 166)	**Anxiety**	−.289	0.035	−0.157	−0.020	**.011**
NA	NA	NA	**Depression**	.008	0.039	−0.074	0.080	.941
NA	NA	NA	**Sleep**	−.028	0.024	−0.053	0.040	.778
Silhouettes	0.081	4.91 (3, 168)	**Anxiety**	−.269	0.098	−0.428	−0.040	**.018**
NA	NA	NA	**Depression**	.001	0.110	−0.217	0.218	.995
NA	NA	NA	**Sleep**	−.023	0.066	−0.146	0.116	.821
Copy task	0.143	9.34 (3, 168)	**Anxiety**	−.205	0.159	−0.611	0.016	.062
NA	NA	NA	**Depression**	.075	0.178	−0.217	0.486	.451
NA	NA	NA	**Sleep**	−.261	0.107	−0.501	−0.079	**.007**
Copy task time	0.009	0.512 (3, 168)	**Anxiety**	.058	2.07	−3.07	5.12	.621
NA	NA	NA	**Depression**	.042	2.33	−3.69	5.50	.699
NA	NA	NA	**Sleep**	.005	1.40	−2.69	2.83	.959
Coding	0162	10.81 (3, 168)	**Anxiety**	−.058	0.37	−0.93	0.53	.590
NA	NA	NA	**Depression**	−.128	0.42	−1.36	0.28	.197
NA	NA	NA	**Sleep**	−.270	0.25	−1.20	−0.21	**.005**
Symbol search	0.080	4.85 (3, 168)	**Anxiety**	−.102	0.23	−0.67	0.25	.368
NA	NA	NA	**Depression**	−.020	0.26	−0.57	0.46	.845
NA	NA	NA	**Sleep**	−.190	0.16	−0.61	0.01	.059
Perception time	0.177	12.03 (3, 168)	**Anxiety**	.248	0.228	0.078	0.980	**.022**
NA	NA	NA	**Depression**	.077	0.256	−0.304	0.708	.431
NA	NA	NA	**Sleep**	.149	0.154	−0.061	0.547	.116
Visual scanning	0.126	8.04 (3, 168)	**Anxiety**	.000	0.17	−0.33	0.33	1.00
NA	NA	NA	**Depression**	.205	0.19	0.01	0.74	**.043**
NA	NA	NA	**Sleep**	.196	0.11	0.00	0.44	**.046**
RTIFMRT	0.032	1.83 (3, 168)	**Anxiety**	.043	1.17	−1.88	2.75	.712
NA	NA	NA	**Depression**	.109	1.31	−1.24	3.94	.307
NA	NA	NA	**Sleep**	.052	0.791	−1.16	1.96	.615
RTIFMMT	0.046	2.68 (3, 168)	**Anxiety**	.079	1.70	−2.18	4.53	.492
NA	NA	NA	**Depression**	.022	1.91	−3.36	4.18	.831
NA	NA	NA	**Sleep**	.137	1.14	−0.717	3.81	.179
Number sequencing	0.066	3.96 (3, 168)	**Anxiety**	.034	0.297	−0.496	0.675	.764
NA	NA	NA	**Depression**	.102	0.333	−0.331	0.983	.329
NA	NA	NA	**Sleep**	.157	0.200	−0.082	0.707	.120
Letter sequencing	0.090	5.53 (3, 168)	**Anxiety**	−.006	0.569	−1.15	1.09	.959
NA	NA	NA	**Depression**	.193	0.638	−0.056	2.46	.061
NA	NA	NA	**Sleep**	.150	0.383	−0.178	1.33	.133
Number-letter switching	0.167	11.21 (3, 168)	**Anxiety**	.114	1.16	−1.06	3.51	.292
NA	NA	NA	**Depression**	−.001	1.30	−2.57	2.56	.995
NA	NA	NA	**Sleep**	.325	0.782	1.13	4.21	**<.001**
Inhibition	0.092	5.63 (3, 168)	**Anxiety**	−.112	0.635	−1.88	0.625	.324
NA	NA	NA	**Depression**	.184	0.719	−0.141	2.69	.077
NA	NA	NA	**Sleep**	.242	0.430	0.193	1.88	**.016**

This table presents the results of linear and multiple regression analyses assessing the impact of education, anxiety (*HADA* scores), depression (*HADD* scores), and sleep (*ISI* scores) on motor and cognitive test performance. The table includes *R*^2^ values, *F* values with degrees of freedom (*df*), standardized regression coefficients (*β*), *SEs*, 95% *CIs* for *β* and *P*-values. NA, not available.

Bold values indicate statistically significant differences.

### Psychostimulant effects on anxiety, depression, and sleep

Table 1 shows the distribution of demographics and clinical variables. A Kruskal-Wallis analysis showed that there were small but significant differences in anxiety, depression, and sleep when comparing all groups. Significant differences in anxiety were observed between controls and U-ADHD (*P* = .000) and between controls and M-ADHD (*P*<.001) and between M-ADHD and U-ADHD (*P* = .018). Significant differences in depression were observed between controls and U-ADHD (*P*<.001) and between controls and M-ADHD (*P*<.001) and as well between M-ADHD and U-ADHD (*P* = .050). Significant differences in sleep scores were observed between controls and U-ADHD (*P*<.001) and between controls and M-ADHD (*P*<.001).

## Discussion

Our results show that both M-ADHD and U-ADHD performed worse than controls on almost all measures of motor and cognitive functions. No overall differences were observed between the M-ADHD and U-ADHD. However, significant differences emerged only when analyses were stratified by disorder severity. Patients with moderate-severe U-ADHD showed more deficits in sensorimotor functions, visual perception, attention and processing speed, as well as partially working memory compared to the M-ADHD. Those with mild U-ADHD displayed lower performances on motor speed, visual scanning, and movement time compared to controls. However, on several tests, the M-ADHD performed better than the U-ADHD group. These findings are consistent with previous literature indicating that medication improves certain cognitive and motor outcomes in ADHD, particularly for attention, processing speed, and visuospatial skills.^55–58^ This suggest that ADHD medication offers benefits for sensorimotor, visuospatial functions, attention and processing speed, with the extent of these benefits varying according to the severity of the disorder.

These results indicate that, compared to controls and M-ADHD, patients with U-ADHD show deficits across several functions, with the deficits being particularly pronounced in those with higher severity. When comparing between the patient group, higher symptom severity is associated with greater impairment. Specifically, deficits were observed in cerebellar and motor functions related to visuomotor adaptation and motor learning. Visuospatial perception and abilities were also affected, with differences evident in tasks measuring visuospatial processing and visual perception. Attention and processing speed were slower in U-ADHD, particularly in psychomotor and RTI components. Executive functions, such as visuomotor speed and partially working memory, showed impairments only in U-ADHD patients with moderate to severe severity. The pattern of deficits aligns with theoretical models that implicate cerebellar, frontoparietal, and basal ganglia networks in ADHD-related cognitive and motor dysfunction.^59–62^ Overall, the findings highlight that severity level influences the extent of cognitive and motor deficits in ADHD groups.

While both M-ADHD and U-ADHD performed worse than controls overall, differences between these groups emerged only when accounting for disorder severity. Patients with moderate to severe U-ADHD showed slower RTIs, lower accuracy and motor speed, as well as greater deficits in sensorimotor, visuospatial, attention, processing speed, and partially working memory functions compared with moderate to severe M-ADHD. However, these findings should be interpreted with caution, as the cross-sectional and non-randomized design does not allow medication effects to be disentangled from clinical characteristics and severity-related factors.

When interpreting differences between ADHD groups, it is essential to consider the influence of medication and comorbid conditions. Our regression analysis showed that only anxiety and sleep had a small but significant effect on test performance, while depression did not. These findings contrast with several previous studies suggesting that psychiatric symptoms, including depression, anxiety, and sleep problems, can meaningfully impact cognitive performance.^63–65^ The discrepancy may be due to differences in sample characteristics, assessment methods, or the relatively low severity of symptoms observed in our cohort with more than 60% classified as mild. Additionally, the wide variety of medications used in our sample makes it difficult to determine their specific impact on cognitive outcomes. From a theoretical perspective, anxiety might selectively affect attention and processing speed due to increased cognitive load and stress-related interference, whereas mild depressive symptoms or sleep disturbances may not exert measurable effects in this sample.^66^ Larger, more homogeneous studies are needed to clarify how these factors interact and to better understand the role of psychiatric symptoms in cognitive performance. Anxiety, depression, and sleep disorders are frequently present alongside ADHD, and widespread medication use further complicates isolating the effects of the core neuropsychiatric disorder. This complexity aligns with prior research that highlights the significant variability in psychiatric comorbidities among individuals with neuropsychiatric disorders.^67^ Significant differences in anxiety, depression, and sleep scores were observed across the groups. Both M-ADHD and U-ADHD participants reported clinically significant higher levels of anxiety (≥8) and sleep (≥7) compared to controls, with the U-ADHD showing the highest levels overall. Similarly, depression scores were elevated in both ADHD groups relative to controls, with the U-ADHD reporting slightly more severe symptoms than the medicated group. Sleep quality was also poorer among individuals with ADHD, regardless of medication status, when compared to the control group.^68^^,^^69^

The severity of the disorder and the symptoms present at the time of diagnosis can affect the findings. Previous research supports that psychostimulants effectively reduce ADHD symptoms.^13^ This suggests that while psychostimulants primarily target core ADHD symptoms, they may also provide modest improvements in sensorimotor and cognitive function. Moreover, our results contribute to the broader literature by emphasizing that the interaction between disorder severity, medication status, and psychiatric comorbidities determines the magnitude of observed cognitive deficits, consistent with multi-factorial models of ADHD.^67^^,^^70^^,^^71^ Additionally, many of the tested variables showed large effect sizes, indicating that these performance differences are clinically meaningful.

## Limitations

In the analysis, we did not consider medication dosages or duration of medication use. We did not control serum levels of active psychostimulants, which may vary with body weight, age, gender, metabolism, and timing of testing. A limitation of the present study is the absence of quantitative measures of ADHD symptom severity, both before and after pharmacological treatment, which prevented a longitudinal evaluation of symptom change. Furthermore, as treatment optimization was based on routine clinical practice rather than standardized rating scales, we cannot determine whether patients had achieved full symptomatic remission at the time of testing.

The relatively small sample size is another limitation, particularly within groups of moderate and severe disorder severity. This reduces statistical power and an increased risk that meaningful effects go undetected. Bonferroni correction strengthens the validity of positive findings, it also increases the risk of type II errors, possibly masking subtle but clinically relevant effects. Given the cross-sectional and non-randomized design, medication effects cannot be disentangled from clinical and severity-related factors. A cross-sectional study on drugs can only detect associations at a single point in time, without establishing causality or temporal sequence. It is also prone to selection bias, confounding, and cannot capture rare or long-term effects.

## Conclusion

Given the cross-sectional and non-randomized design, these findings should be interpreted as associations, as medication effects cannot be disentangled from clinical and severity-related factors. M-ADHD and U-ADHD showed significantly poorer performance than controls across all assessed domains. When disorder severity was not taken into account, no significant differences were observed between M-ADHD and U-ADHD. However, severity-stratified analyses revealed distinct patterns of cognitive outcomes. Overall, the full test battery showed clear differences between controls and ADHD, but minimal differences between U-ADHD and M-ADHD. Specifically, individuals with moderate-severe U-ADHD demonstrated greater impairments in sensorimotor functions, visual perception, attention and processing speed, and partial working memory compared with the M-ADHD group, whereas participants with mild U-ADHD exhibited lower motor speed, visual scanning, and movement time compared to controls.

## Future research

Though our study is based on a relatively large sample, the subgroups were small, and we only examined adults. Future research should aim to test a larger number of individuals with different levels of severity and extend the sample to include children. This could yield more nuanced insights into how different subgroups of ADHD patients respond to psychostimulant medication. Furthermore, exploring the relationship between severity index and psychostimulant medication status could improve the understanding of the effect of medication and optimization of dosages for ADHD patients based on their functioning level, facilitating individualized care. Future studies should investigate whether combining objective cognitive and motor assessments may enhance diagnostic accuracy for ADHD.

## Supplementary Material

SUPPLEMENT_1_pyag013

SUPPLEMENT_2_pyag013

SUPPLEMENT_3_pyag013

SUPPLEMENT_4_pyag013

## Data Availability

The data that support the findings are available upon reasonable request from the corresponding author.
