# Supplementary material for: Psychostimulant effects on motor and cognitive function in adults attention deficit hyperactivity disorder
Source: Int J Neuropsychopharmacol. 2026 Mar 26;29(4):pyag013. doi: 10.1093/ijnp/pyag013 (PMC13130065; doi:10.1093/ijnp/pyag013)
Supplement: SUPPLEMENT_1_pyag013 [file supplement_1_pyag013.pdf]

SUPPLEMENT 1 - Comorbidies

The data describes various diagnoses or conditions observed among individuals in the dataset. The conditions and their respective frequencies are as follows:

Medicated-ADHD (n=40)

- **Arthritis:** Observed in 1 case.
- **Atrioventricular Block:** Observed in 1 case.
- **Bipolar II Disorder:** Observed in 1 case.
- **Deficits in Attention, Motor Control, and Perception (DAMP):** Observed in 1 case.
- **Dyscalculia:** Observed in 1 case.
- **Dyslexia:** Observed in 1 case.
- **Emotionally Unstable Personality Disorder (EUPD):** Observed in 2 cases.
- **Gender Dysphoria:** Observed in 1 case.
- **Mental and Behavioral Disorders Due to Opioid Dependence Syndrome:** Observed in 1 case.
- **Receptive Language Disorder:** Observed in 1 case.
- **Spastic Diplegia Cerebral Palsy:** Observed in 1 case.
- **Spinal Stenosis:** Observed in 1 case.

Unmedicated-ADHD (n=52)

- **Deficits in Attention, Motor Control, and Perception (DAMP):** Observed in 1 case.
- **Developmental Expressive Language Disorder:** Observed in 2 cases.
- **Diabetes Type II:** Observed in 1 case.
- **Dyslexia:** Observed in 4 cases.
- **Expressive Language Disorder:** Observed in 2 cases.
- **Heart Failure (previous):** Observed in 1 case.
- **Hypopituitarism:** Observed in 1 case.
- **Idiopathic Epilepsy:** Observed in 1 case.
- **Mental and Behavioral Disorders Due to Opioid Dependence Syndrome:** Observed in 1 case.
- **Obesity:** Observed in 1 case.
- **Personal History of Traumatic Brain Injury:** Observed in 1 case.
- **Sensorineural Hearing Loss:** Observed in 1 case.
- **Unspecified Bipolar Disorder:** Observed in 1 case.
- **Unspecified Social Phobia:** Observed in 1 case.

Control group (n=80)

- **Arthritis:** Observed in 1 case.
- **Asthma:** Observed in 1 case.
- **Atopic Dermatitis (unspecified):** Observed in 1 case.
- **Crohn’s Disease:** Observed in 1 case.
- **Gilbert's syndrome:** Observed in 1 case.
- **History of Hypophysis Tumor (operated):** Observed in 1 case.
- **Irritable Bowel Syndrome:** Observed in 2 cases.
- **Menopausal and Female Climacteric States:** Observed in 1 case.
- **Premenstrual Tension Syndrome:** Observed in 1 case.
- **Psoriatic Arthritis:** Observed in 1 case.
- **Recurrent Depressive Disorder (in remission):** Observed in 1 case.
- **Ulcerative Colitis:** Observed in 1 case.
