# Supplementary material for: Psychostimulant effects on motor and cognitive function in adults attention deficit hyperactivity disorder
Source: Int J Neuropsychopharmacol. 2026 Mar 26;29(4):pyag013. doi: 10.1093/ijnp/pyag013 (PMC13130065; doi:10.1093/ijnp/pyag013)
Supplement: SUPPLEMENT_2_pyag013 [file supplement_2_pyag013.pdf]

SUPPLEMENT 2 - Medication Dosages and Active Ingredients

This supplement categorizes the medications by their active ingredients and provides a quantification of how often each medication and dosage appear in the dataset. It helps give an overview of the medications administered and their frequencies across the sample.

Medicated-ADHD (n=40)

Active Ingredient	Frequency	Dosage (mg)	Total Count (Dosage)
**Bupropion**	1	150	1
**Dexamphetamine**	2	5	2
	2*	5	1
	1*	20	1
	1	30	5
	1	40	5
	1	50	1
	1	60	4
**Duloxetine**	1	60	1
**Enalapril**	1	20	1
**Fluoxetine**	2	20	3
	3	20	1
**Guanfacine**	1	2	1
**Hydroxyzine**	1	12.5	1
**Lamotrigine**	1	100	2
	1	200	1
**Levothyroxine**	1	50	1
**Melatonin**	1	3	1
	1	5	7
	1	7.5	2
**Methylphenidate**	1	18	5
	1	20	1
	1	27.5	1
	2	30	1
	1	30	2
	1	36	5
	1	40	2
	1	45	1
	1	54	7
**Mirtazapine**	1	5	2
	1	7.5	2
**Propiomazine**	1	10	1
	1	25	2
**Propranolol**	2	40	1
**Reboxetine**	1	4	1
**Sertraline**	1	50	5
	2	100	1
	1	120	1

**Testosterone**	1	1000	1
**Venlafaxine**	1	75	3
	1	225	1
**Zolpidem**	1	10	1

Notes: *= the person assumes the medication on demand; **= One time every three months.

Unmedicated-ADHD (n=52)

Active Ingredient	Frequency	Dosage (mg)	Total Count (Dosage)
**Acetylsalicylic acid**	1	75	1
**Alimemazine**	1*	20	1
**Atorvastatin**	1	80	1
**Bisoprolol**	1	5	1
**Buprenorphine**	1**	125	1
**Bupropion**	1	150	1
**Citalopram**	1	40	1
**Escitalopram**	2	10	1
**Fluoxetine**	2	20	1
**Lamotrigine**	1	150	2
**Levothyroxine**	1	137.5	1
	1	200	1
**Melatonin**	1	3	1
	1	10	1
**Propiomazine**	1	25	1
**Propranolol**	1*	10	1
**Ramipril**	1	10	1
**Sertraline**	1	50	1
	1	100	1
**Spironolactone**	1	12.5	1
**Venlafaxine**	1	37.5	1
	1	150	1
	1	225	1
**Vortioxetine**	1	10	1

Notes: *= the person assumes the medication on demand; **= One time per month.

Control group (n=80)

Active Ingredient	Frequency	Dosage (mg)	Total Count (Dosage)
**Adalimumab**	2**	40	1
**Allergy pill**	1*	-	1
**Azathioprine**	1	-	1
**Bisoprolol**	1	2.5	1
**Citalopram**	1	30	1
**Enalapril**	1	5	1
**Isotretinoin**	1	-	1
**Levothyroxine**	1	75	1
**Losartan**	1	10	1
**Melatonin**	1*	8	1
**Pregabalin**	2	150	1
**Progesterone**	1	-	1
**Sertraline**	1	50	1
	1	100	1
	1	150	1
**Sumatriptan**	1*	-	1

Notes: *= the person assumes the medication on demand; **= Two times per month; -= Dosage not listed because the person does not remember the specific amount.
