# Supplementary material for: Psychostimulant effects on motor and cognitive function in adults attention deficit hyperactivity disorder
Source: Int J Neuropsychopharmacol. 2026 Mar 26;29(4):pyag013. doi: 10.1093/ijnp/pyag013 (PMC13130065; doi:10.1093/ijnp/pyag013)
Supplement: SUPPLEMENT_3_pyag013 [file supplement_3_pyag013.pdf]

SUPPLEMENT 3 - Cerebellar Tests

Cerebellar tests

Finger Tapping.

This test measures sensorimotor synchronization and the ability to maintain rhythm and can be used to assess motor control and the integrity of the neuromuscular system [39]. We evaluated isochronous serial interval production, that has been linked to cerebellar function [40, 41]. The participant must synchronize taps on the space bar to a rhythmic sound (synchronization phase) and continue pressing the space bar at the same pace when the rhythmic sound stops (production phase). This procedure was repeated five times. Each repetition included 15 paced intervals followed by 70 self-paced intervals. The inter-onset interval (IOI) was 524 ms. The mean inter-tap interval and two measures of variance (drift and local) during the production phase were used for the analysis. Response IOIs shorter than 400 ms and longer than 650 ms were excluded from the analyses. The variables selected for the analysis were production mean (ms), local (ms), and drift (ms).

Prism Adaptation.

This test measures sensorimotor coordination following changes in visual input. Previous studies demonstrate a relationship between performance in prism adaptation and cerebellar function [42, 43]. Participants stood at an arm's length from a measuring tape attached to the wall. On each trial, the participant was instructed to look at the measuring tape and identify the midpoint of the measuring tape, then close their eyes and point to the target. While still pointing and standing in the same place, the participants opened their eyes to see where they had pointed. This procedure was repeated 10 times without the glasses. For the next 10 trials, the participants wore prism glasses that displaced the visual field laterally by 15 degrees. Wearing the prisms usually results in a

pointing error that gradually decreases as the participant adapts to the new visuomotor relationship. In the last ten trials (21–30), the glasses were taken off again. Removing the glasses typically results in a pointing error in the direction opposite to the error that occurred when putting the prisms on – often to the surprise of the participant. On each trial, the test administrator wrote down the error in centimeters. For the analysis we examined the error on the first trial after putting the glasses on (trial 11), the error on the first trial after taking the glasses off (trial 21) and the average absolute error across all 30 trials.
