# Supplementary material for: Psychostimulant effects on motor and cognitive function in adults attention deficit hyperactivity disorder
Source: Int J Neuropsychopharmacol. 2026 Mar 26;29(4):pyag013. doi: 10.1093/ijnp/pyag013 (PMC13130065; doi:10.1093/ijnp/pyag013)
Supplement: SUPPLEMENT_4_pyag013 [file supplement_4_pyag013.pdf]

SUPPLEMENT 4 - Psychometric Properties

We calculated the concurrent validity for the exploratory neuropsychological tests.

The concurrent validity between ***Motor Speed***, subtest of *Trail Making Test* of *Delis-Kaplan Executive Function System (D-KEFS)* and ***Motor Time***, subtest of *Bender-Gestalt II* in our sample was ρ=0.55 (p<.001) and the split-half reliability was rs=0.71. The concurrent validity between the ***Perception Time***, subtest of *Bender-Gestalt II* and ***Symbol Search***, subtests of *Wechsler Adult Intelligence Scale (WAIS-IV)* in our sample was r=-0.64 (p<.001), and the split-half reliability was rs=-0.78.

The concurrent validity between ***Copy Task*,** *Visual Reproduction II (VR-II)* of Wechsler *Memory Scale III (WMS-III)* and ***Cube Analysis***, subtests of *Visual Object and Space Perception Battery (VOSP)* in our sample was ρ=0.31 (p<.001). The concurrent validity between ***Copy Task***, *Visual Reproduction II (VR-II)* of *Wechsler Memory Scale III (WMS-III)* and ***Silhouettes***, subtests of *Visual Object and Space Perception Battery (VOSP)* was ρ=0.26 (p<.001).
